# The mediating role of emotional intelligence in the relationship between physical education and sports teachers’ mindfulness and psychological resilience

**DOI:** 10.3389/fpsyg.2026.1815783

**Published:** 2026-05-29

**Authors:** Mücahit Dursun, Emirhan Kan, Hasan Ceyhun Can, Yunus Emre Yarayan, Erdoğan Tozoğlu, Kenan Şebin, Yusuf Buzdağlı

**Affiliations:** 1 Faculty of Sports Sciences, Selcuk University, Konya, Türkiye; 2 Faculty of Sports Sciences, Atatürk University, Erzurum, Türkiye; 3 Faculty of Sport Sciences, Istanbul University-Cerrahpaşa, Avcılar, Türkiye; 4 School of Physical Education and Sports, Siirt University, Siirt, Türkiye; 5 Turkish Curling Federation, Ankara, Türkiye; 6 Faculty of Sports Sciences, Erzurum Technical University, Yakutiye, Türkiye

**Keywords:** emotional intelligence, mindfulness, physical education, psychological resilience, sport teaching

## Abstract

**Introduction:**

Although associations among mindfulness, emotional intelligence, and psychological resilience have been reported in previous research, evidence clarifying the indirect pathways among these variables within specific occupational contexts, particularly among physical education and sports teachers, remains limited. Therefore, this study examined the associations between teachers’ dispositional mindfulness and psychological resilience and investigated the mediating role of emotional intelligence in this relationship.

**Methods:**

Using a cross-sectional correlational design, data were collected from 366 physical education and sports teachers working in public secondary and high schools in Erzurum province, Türkiye. Mindfulness was assessed using the Mindful Attention Awareness Scale (MAAS), emotional intelligence using the Trait Emotional Intelligence Questionnaire–Short Form (TEIQue-SF), and psychological resilience using the Brief Resilience Scale (BRS). Confirmatory factor analyses were conducted to evaluate the measurement properties of the instruments. Indirect effects were tested using PROCESS Model 4 with bootstrap resampling (5,000 samples), while controlling for years of professional experience. In addition, a complementary parcel-based structural equation modeling (SEM) analysis was conducted to examine the robustness of the mediation findings under a latent-variable framework.

**Results:**

The results indicated that dispositional mindfulness was positively associated with psychological resilience. Emotional intelligence demonstrated a significant mediating effect in the relationship between mindfulness and resilience. While the composite-score analysis suggested partial mediation, the complementary SEM analysis supported full mediation.

**Conclusion:**

These findings suggest that higher levels of mindful attention and awareness are associated with greater psychological resilience, primarily through emotional intelligence–related capacities. Given the cross-sectional and self-report nature of the data, the findings should be interpreted as associative rather than causal. Overall, the results highlight the potential relevance of mindfulness- and emotion-focused approaches in supporting the psychological functioning of physical education and sports teachers.

## Introduction

Maintaining psychological resilience in the face of complex stressors and constantly changing conditions brought about by modern social life is of great importance. Psychological resilience refers to individuals’ capacity to adapt, recover, and grow when confronted with difficulties, trauma, and stressful life events, and is increasingly conceptualized as a dynamic and multifactorial process of successful adaptation to adversity (Nadifi et al., 2026). Individuals with high levels of psychological resilience are better able to cope with adverse experiences and maintain functional well-being under challenging circumstances. Within the teaching profession, psychological resilience has been identified as a key factor in coping with occupational stress, maintaining job satisfaction, and preventing burnout (Reintjes et al., 2025). For example, Kartol et al. (2024) reported that individuals with higher emotional intelligence demonstrated stronger stress-management capacities and more adaptive psychological responses under challenging conditions, supporting the association between emotional intelligence and resilience-related functioning.

Mindfulness has recently gained popularity as one of the elements that may be linked to psychological resilience. Stoyanova et al. (2025) defined mindfulness as ‘paying attention to what is happening in the moment without judgment’; this means that people are aware of their surroundings and their inner feelings. Mindfulness can be conceptualized both as a state and as a trait. State mindfulness refers to momentary awareness and attention to present experiences, whereas trait mindfulness reflects a relatively stable dispositional tendency to be attentive and aware in daily life. In the present study, mindfulness was conceptualized as trait mindfulness, consistent with the use of the Mindful Attention Awareness Scale, which assesses dispositional mindful attention and awareness. Systematic reviews have consistently demonstrated that mindfulness practices significantly reduce symptoms of stress, anxiety, and depression, while enhancing overall psychological health (Zhang et al., 2023). In recent years, mindfulness has been increasingly examined within educational contexts, particularly in relation to teachers’ well-being, stress management, and classroom functioning (Pathak et al., 2025).

Empirical studies have consistently reported positive associations between mindfulness and psychological resilience. Meta-analytic findings further indicate that mindfulness-based interventions are linked to improvements in resilience-related outcomes (Zhang et al., 2023). However, the underlying psychological mechanisms explaining this relationship remain insufficiently understood. This gap is particularly relevant for physical education and sports teachers, whose professional roles involve both physical instruction and continuous socio-emotional interaction with students in dynamic environments. Accordingly, the first hypothesis of the present study is formulated as follows:

*H1*: Mindfulness positively affects psychological resilience.

An important factor that helps explain this relationship is the concept of emotional intelligence. According to Lv et al. (2024), emotional intelligence is ‘the ability to perceive, understand, use, and regulate one’s own and others’ emotions’ and is vital to an individual’s social and personal functioning. People with high emotional intelligence can respond more amicably when faced with difficulties and cope more skilfully with stressful situations (Collado-Soler et al., 2023). Emotional intelligence has also been widely studied in educational contexts, where it is associated with teachers’ classroom management, interpersonal effectiveness, and professional well-being (Valente et al., 2019).

Studies using structural equation modeling have demonstrated strong positive relationships between mindfulness and emotional intelligence (Martínez-Pérez et al., 2023). These findings suggest that emotional intelligence may play a mediating role in the relationship between mindfulness and psychological resilience.

Theoretically, mindfulness exercises are expected to systematically increase people’s emotional awareness and regulation capacities (Guendelman et al., 2017). By strengthening the core elements of emotional intelligence, this developmental process may be associated with psychological resilience and help individuals develop more effective coping mechanisms when faced with challenges. According to this theoretical framework, the formulation of the second hypothesis is as follows:

*H2*: Mindfulness positively affects emotional intelligence.

The literature indicates that mindfulness has a positive effect on emotional intelligence and psychological resilience (Martínez-Pérez et al., 2023). Given these findings, it is thought that developed emotional intelligence may mediate a significant portion of mindfulness’s effect on psychological resilience. According to Extremera and Rey (2016), new research suggests that emotional intelligence may serve as a mediator between related psychological systems. This experimental support has led to the development of the following third hypothesis:

*H3*: Emotional intelligence positively affects psychological resilience.

However, there are relatively few studies that examine the mediating function of emotional intelligence within an integrative analytical framework that considers mindfulness, emotional intelligence, and psychological resilience simultaneously (Schutte and Meynadier, 2024). Existing research has predominantly focused on bivariate or pairwise associations among these variables, while the psychological mechanisms underlying their interrelationships have received limited empirical attention. Despite the growing body of research on these constructs, studies specifically focusing on physical education and sports teachers remain limited. Therefore, examining the associations among these variables within this specific professional context may provide more nuanced insights. This gap indicates a need for mechanism-oriented mediation models that clarify how mindfulness may translate into psychological resilience through emotional processes. Accordingly, the fourth hypothesis of the present study is formulated as follows:

*H4*: Emotional intelligence mediates the role of mindfulness on psychological resilience

In addition, teachers’ years of professional experience may shape both mindfulness-related competencies (e.g., attentional control and emotion regulation) and psychological resilience through accumulated classroom exposure and coping routines. To reduce potential confounding due to professional tenure, years of professional experience were included as a covariate in the mediation model.

To understand the nature of the relationships between variables and their potential mediating associations, a correlational research design was selected for this study. Correlational approaches are widely used in psychological and educational research to examine theoretically grounded associations among naturally occurring psychological constructs without experimental manipulation (Christensen et al., 2015). In addition, mediation-based analytical frameworks are considered appropriate for exploring potential explanatory mechanisms underlying observed associations among variables within non-experimental designs (Hayes, 2018). The interactions between complex psychological constructs such as emotional intelligence, mindfulness, and psychological resilience may not be adequately captured through short-term experimental manipulations alone. Therefore, the present design aimed to examine theoretically meaningful relational patterns based on participants’ existing levels of these psychological characteristics.

The primary aim of this study is to examine whether emotional intelligence is associated with the relationship between teachers’ mindfulness and psychological resilience within a theory-driven mediation framework. The research findings are expected to contribute to the development of evidence-based strategies aimed at enhancing the effectiveness of mindfulness-based interventions in psychological counseling and psychotherapy practices, as well as informing preventive intervention initiatives designed to strengthen teachers’ psychological resilience.

## Method

### Study model

**Figure 1:**
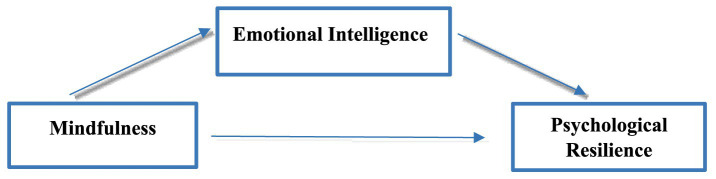
Research model.

This study examines the effect of Physical Education and Sports Teachers’ mindfulness on their psychological resilience and the mediating role of emotional intelligence in this effect. The research was conducted using a quantitative research method, specifically a correlational survey model aimed at revealing the relationship between variables (Christensen et al., 2015). The literature highlights the importance of identifying mediating variables to better understand the mechanisms underlying observed relationships and to advance theoretical explanations in psychological research (Hayes, 2018). Accordingly, emotional intelligence was specified as a mediator in the proposed model, while years of professional experience were included as a control variable to account for potential confounding effects related to professional tenure. Years of professional experience were included as a control variable based on prior theoretical considerations rather than bivariate correlations. In this context, the theoretical model tested in the study is presented in Figure 1.

### Research group

An *a priori* power analysis was conducted using G*Power 3.1 software to determine the minimum required sample size for regression-based analyses. Based on a significance level of 0.05, a statistical power of 0.95, and a medium effect size (*f*^2^ = 0.15) for the main regression models, the minimum required sample size was estimated to be approximately 120 participants. In line with recommendations regarding potential data loss or missing responses (Suresh and Chandrashekara, 2012), the sample size was increased beyond the minimum requirement. Accordingly, data from a total of 366 participants were included in the study. The sample consisted of 170 female participants (*M* = 34.94, SD = 7.38) and 196 male participants (*M* = 36.30, SD = 8.91). The mean professional experience was 10.14 years (SD = 6.86) for female participants and 12.12 years (SD = 9.26) for male participants. Participants were recruited using a convenience sampling strategy through institutional contacts and professional networks. All participants were actively working as physical education and sports teachers in public secondary and high schools. Participation was voluntary, and no financial incentives were provided.

### Inclusion and exclusion criteria and professional context

Participants were eligible for inclusion if they were actively employed as Physical Education and Sports Teachers in public secondary or high schools during the data collection period, voluntarily agreed to participate, and completed all study measures. Individuals working in non-teaching or administrative positions without active teaching responsibilities, those who declined participation, or those who provided incomplete or inconsistent responses were excluded from the study. These criteria were established to ensure greater homogeneity in terms of professional responsibilities, workload, and educational context.

### Data collection procedure

Data were collected over approximately 4 weeks in accordance with participants’ teaching schedules and institutional permissions. Participants were recruited from multiple public schools across different regions through institutional contacts and professional networks. A total of 560 Physical Education and Sports Teachers were contacted, and 366 agreed to participate, for a participation rate of approximately 65.4%.

Before participation, all teachers were informed about the purpose and procedures of the study and completed a personal information form, followed by the measurement instruments. No time restrictions were imposed during questionnaire completion.

Data collection was conducted using both online and face-to-face procedures. A portion of the participants completed the questionnaires online via Google Forms, while the remaining participants completed the same instruments in person under researcher’s supervision. To ensure procedural consistency, all participants received identical instructions and completed the same measurement instruments using the same item order and response format regardless of the administration mode. This approach was adopted to maintain consistency across data-collection conditions and to minimize procedural differences between online and face-to-face applications.

### Data collection tools

#### Trait emotional intelligence questionnaire-short form

The Turkish adaptation of the TEIQue-SF originally developed by Konstantinos V. Petrides and Adrian Furnham and adapted into Turkish by Deniz et al. (2013) was used in this study. The scale consists of 20 items and assesses global trait emotional intelligence. A confirmatory factor analysis (CFA) was conducted to evaluate the scale’s construct validity. The results demonstrated acceptable overall model fit [*χ*^2^/df = 3.37, GFI = 0.90, CFI = 0.87, RMSEA = 0.079, 90% CI (0.065, 0.092), SRMR = 0.048]. Although the CFI value was slightly below the conventional 0.90 criterion suggested by Li-tze Hu and Peter M. Bentler, the fit indices collectively indicated an acceptable measurement structure.

Inspection of the modification indices revealed localized residual covariances between several item pairs (Items 13–15, 1–3, and 17–20). Correlated error terms were therefore specified between these pairs based on both statistical evidence and conceptual similarity among the items, particularly regarding closely related aspects of emotional perception and regulation. No modifications were introduced between conceptually unrelated dimensions.

The scale demonstrated acceptable internal consistency (Cronbach’s *α* = 0.70). In addition, the composite reliability (CR) coefficient was 0.89, indicating satisfactory construct reliability. However, the average variance extracted (AVE) value was 0.34 [90% CI (0.31, 0.39)], which remained below the conventional 0.50 threshold. Therefore, although the relatively high CR value partially supported the construct’s reliability, the measure’s convergent validity should be interpreted cautiously. To reduce the potential influence of measurement error associated with these psychometric limitations, the mediation model was evaluated using both regression-based PROCESS analysis and latent-variable structural equation modeling (SEM). Because SEM explicitly separates measurement error from latent construct variance, this complementary approach provided a more rigorous assessment of the robustness of the indirect effect estimates.

#### Mindful attention awareness scale

The MAAS was originally developed by Kirk Warren Brown and Richard M. Ryan and later adapted into Turkish by Özyeşil et al. (2011). The scale consists of 15 items rated on a five-point Likert scale and assesses dispositional mindful attention and awareness within a unidimensional structure.

In the present study, confirmatory factor analysis (CFA) was conducted to evaluate the factorial validity of the scale. The results indicated acceptable model fit [*χ*^2^/df = 2.49, GFI = 0.92, CFI = 0.92, RMSEA = 0.064, 90% CI (0.053, 0.075), SRMR = 0.050]. Examination of the modification indices revealed localized residual covariances between Items 7–10 and Items 12–15. Correlated error terms were therefore specified between these item pairs based on both statistical evidence and conceptual similarity, as the items reflected closely related aspects of attentional awareness and present-moment focus. No modifications were introduced between conceptually unrelated items.

The scale demonstrated high internal consistency in the present sample (Cronbach’s *α* = 0.86). In addition, the composite reliability (CR) coefficient was 0.86, supporting satisfactory construct reliability. The average variance extracted (AVE) value was 0.30 (90% CI [0.27, 0.34]), which remained below the conventional 0.50 threshold. Nevertheless, considering the acceptable overall model fit and reliability coefficients, the findings generally supported the adequacy of the MAAS for assessing dispositional mindfulness in the present study.

#### Brief resilience scale

The BRS was originally developed by Smith et al. (2008) and adapted into Turkish by Doğan (2015). The scale consists of 6 items rated on a five-point Likert scale and assesses the ability to recover from stress within a unidimensional structure. Items 2, 4, and 6 are reverse-scored.

In the present study, confirmatory factor analysis (CFA) was conducted to evaluate the factorial validity of the scale. The results demonstrated acceptable model fit [*χ*^2^/df = 4.36, GFI = 0.98, CFI = 0.97, RMSEA = 0.076, 90% CI (0.060, 0.135), SRMR = 0.047]. Examination of the modification indices revealed localized residual covariances between several item pairs (Items 1–4, 1–3, and 2–3). Correlated error terms were therefore specified between these items based on both statistical evidence and conceptual similarity, as the paired items reflected closely related aspects of resilience-related recovery and coping processes. No modifications were introduced between conceptually unrelated items.

The scale demonstrated satisfactory internal consistency in the present sample (Cronbach’s *α* = 0.77). In addition, the composite reliability (CR) coefficient was 0.75, indicating acceptable construct reliability. The average variance extracted (AVE) value was 0.37 [90% CI (0.32, 0.43)], which remained below the conventional 0.50 threshold. Although this finding suggests relatively limited convergent validity at the latent construct level, the overall fit indices and reliability coefficients supported the adequacy of the scale for assessing psychological resilience in the present study.

### Overall validity and reliability of the measurement instruments

The psychometric properties of the measurement instruments were evaluated separately within their respective subsections. Confirmatory factor analysis (CFA) results, model fit indices, Cronbach’s alpha coefficients, composite reliability (CR), and average variance extracted (AVE) values were reported and interpreted individually for each scale to avoid repetitive reporting and provide scale-specific psychometric evaluation.

In addition, because some psychometric indicators, particularly the AVE values and convergent validity estimates of the emotional intelligence scale, were comparatively lower, complementary latent-variable structural equation modeling (SEM) analyses were conducted alongside the regression-based PROCESS analyses. By explicitly partitioning measurement error from latent-construct variance, SEM provided a more rigorous evaluation of the robustness and consistency of indirect effect estimates.

### Data analysis

Before conducting the main analyses, missing data and outliers were examined using SPSS 27.0. The initial dataset consisted of 379 participants; however, 13 cases were excluded from the analyses due to multivariate outlier detection based on Mahalanobis distance, resulting in a final sample of 366 participants. Descriptive statistics and distributional properties of the study variables were then evaluated. For emotional intelligence, the skewness and kurtosis values were −0.006 and −0.029, respectively. Mindfulness exhibited skewness and kurtosis values of −0.465 and 0.601, while psychological resilience showed skewness of −0.133 and kurtosis of −0.217. All skewness and kurtosis values fell within the acceptable range of −1 to +1, indicating that the distributions did not substantially deviate from normality (Hair et al., 2014). In addition, visual inspections of histograms and Q–Q plots indicated no substantial deviations from normality.

Linearity among variables was examined using scatter plots, and no significant deviations from linear relationships were observed. Multicollinearity was assessed using tolerance and variance inflation factor (VIF) values, and no multicollinearity problems were detected.

To assess the potential impact of common method bias, Harman’s single-factor test was conducted (Podsakoff et al., 2003). The results indicated that a single factor accounted for 18.61% of the total variance, which is below the commonly referenced threshold of 50%. However, it is important to note that Harman’s single-factor test has been criticized for its limited sensitivity and should not be considered a definitive assessment of common method bias (Podsakoff et al., 2003). Therefore, although the findings suggest that common method bias is not likely to be a dominant issue, its potential influence cannot be completely ruled out.

Following the preliminary analyses, the hypotheses were first tested using PROCESS Macro Model 4 (Hayes, 2018) based on observed composite scores, with years of professional experience included as a covariate. Bootstrap resampling with 5,000 samples was used to estimate indirect effects, and mediation was considered significant when the 95% bias-corrected confidence interval did not include zero.

In the present study, parcel construction procedures varied according to the structural characteristics of each scale. For the MAAS and BRS, parcels were created using a sequential item-grouping approach to preserve the original unidimensional structure of the scales and to obtain balanced indicators. For the TEIQue-SF, a domain-representative parceling strategy based on conceptual similarity and factor loadings was adopted. These approaches were employed to obtain more stable parameter estimates and to reduce model complexity, particularly given the number of observed indicators relative to the sample size. Each latent construct was represented by multiple parcels, and the detailed parcel assignments are provided in the Supplementary materials. The use of parceling is consistent with methodological recommendations suggesting that parceling can improve estimation stability and model fit when constructs demonstrate unidimensional or near-unidimensional structures. In addition, to complement the composite-score approach and to evaluate the proposed relationships under a latent-variable framework, the mediation model was re-tested using structural equation modeling (SEM). For this supplementary SEM analysis, parcel-based latent indicators were employed to obtain a more parsimonious model and reduce parameter burden relative to full item-level estimation. In addition, bootstrap procedures were employed within the SEM framework to estimate the confidence intervals of indirect effects. A total of 5,000 bootstrap resamples were generated, and bias-corrected confidence intervals were obtained. Indirect effects were considered statistically significant when the corresponding confidence intervals did not include zero. All SEM analyses were conducted using AMOS software. This complementary analysis enabled the indirect effect to be examined while explicitly accounting for measurement error. The results of the composite-score PROCESS analysis and the parcel-based latent SEM analysis were then compared to assess the robustness of the mediation findings. All scale scores used in the analyses were calculated as total (sum) scores in accordance with the original scoring procedures of the respective instruments and their Turkish adaptation studies. Because all participants completed the full set of questionnaire items and no item-level missing data remained after the screening procedures, the use of total versus mean scores would not alter the statistical relationships among variables, as mean scores represent only linear transformations of total scores. Therefore, the use of total scores was considered methodologically appropriate and consistent with previous applications of these instruments in psychological and educational research.

Although item-level structural equation modeling provides a more detailed representation of latent constructs, it was not adopted in the present study due to considerations related to model complexity and estimation stability. Given the sample size and the number of observed indicators across constructs, estimating a full item-level model would substantially increase the number of free parameters and reduce estimation precision. Therefore, a parcel-based SEM approach was preferred as a more parsimonious and statistically robust alternative.

## Results

### Correlation analysis and descriptive statistics

Table 1 presents the means, standard deviations, and Pearson correlation coefficients among the study variables. Emotional intelligence was positively and significantly correlated with mindfulness (*r* = 0.40, *p* < 0.01) and psychological resilience (*r* = 0.36, *p* < 0.01). Mindfulness also showed a positive and significant association with psychological resilience (*r* = 0.38, *p* < 0.01). Years of professional experience was not significantly correlated with the main study variables. These findings provide preliminary support for the proposed mediation model.

**Table 1:** Correlations, means, and standard deviations of the study variables.

Variable		1 Years of professional experience	2 Emotional intelligence	3 Mindfulness	4 Psychological resilience	Mean	SD
1. Years of Professional Experience	r	1				11.19	8.28
2. Emotional Intelligence	r	0.093	1			95.38	12.40
3. Mindfulness	r	0.078	0.396^**^	1		60.68	12.55
4. Psychological Resilience	r	0.093	0.360^**^	0.375^**^	1	20.39	4.48

*N* = 366. *p* < 0.01.

It should be noted that all scale scores in the present study were calculated as total (sum) scores based on the respective measurement instruments. Therefore, the reported values reflect the sum of item responses rather than mean scores, and do not fall within the original response ranges of the scales.

Table 2 presents the results of the Hayes PROCESS Macro (Model 4) analysis conducted to test the mediating role of emotional intelligence in the relationship between mindfulness and psychological resilience, with years of professional experience included as a control variable (covariate).

**Table 2:** Results of the mediation analysis examining the role of emotional intelligence in the relationship between mindfulness and psychological resilience.

Predictor variables	Dependent variable: emotional intelligence (M)				Dependent variable: psychological resilience (Y)			
	Coeff (b)	SE	*t*	*p*	Coeff (b)	SE	*t*	*p*
Constant	70.86	3.00	23.61	0.000	5.67	1.69	3.35	0.000
Years of Experience (Control)	0.093	0.072	1.29	0.196	0.026	0.25	1.02	0.308
Mindfulness (X)					0.097	0.018	5.31	0.000
Emotional intelligence (M)					0.089	0.019	4.80	0.000
*R* ^2^	0.16				0.20			
*F* value	34.821				29.375			

Effect summary	Effect (β)	BootSE	*t*	*p*	LLCI	ULCI
Total effect (c)	0.132	0.017	7.59	0.000	0.097	0.166
Direct effect (c′)	0.097	0.018	5.31	0.000	0.061	0.133
Indirect effect (a × b)	0.034	0.008	–	–	0.020	0.050

The results of the first regression model, which examined the explanatory power of the predictor variables (mindfulness and years of professional experience) on the mediator variable (emotional intelligence), indicated that the model was statistically significant (*F* = 34.82, *p* < 0.001). In this model, mindfulness and years of professional experience jointly explained approximately 16% of the variance in emotional intelligence (R^2^ = 0.16). Examination of the regression coefficients revealed that mindfulness positively and significantly predicted emotional intelligence, whereas years of professional experience did not have a statistically significant effect on emotional intelligence (*p* > 0.05).

In the second stage of the analysis, in which the mediator was included in the model, the results showed that mindfulness, years of professional experience, and emotional intelligence together explained approximately 20% of the variance in psychological resilience, and the overall model was statistically significant (*F* = 29.38, *p* < 0.001, *R*^2^ = 0.20). Analysis of the regression coefficients demonstrated that emotional intelligence significantly and positively predicted psychological resilience (*B* = 0.089, *p* < 0.001). Moreover, even after emotional intelligence was included in the model, the direct effect of mindfulness on psychological resilience (c’) remained statistically significant (*B* = 0.097, *p* < 0.001).

To test the significance of the mediation effect, a bootstrap analysis with 5,000 resamples was conducted. The results indicated that the bias-corrected 95% confidence interval for the indirect effect of emotional intelligence (a × b) ranged from 0.020 to 0.050. Because this confidence interval did not include zero, the mediating role of emotional intelligence was confirmed as statistically significant. Furthermore, since the direct effect of mindfulness on psychological resilience remained significant, emotional intelligence was found to play a partial mediating role in this relationship.

Taken together, these findings suggest that mindfulness among physical education and sports teachers not only enhances psychological resilience directly but also strengthens it indirectly by fostering emotional intelligence.

Table 3 presents the parcel-based structural equation modeling results of the proposed mediation model examining the role of emotional intelligence in the relationship between mindfulness and psychological resilience. The overall model demonstrated acceptable fit to the data [*χ*^2^/df = 3.09, CFI = 0.943, TLI = 0.922, GFI = 0.942, AGFI = 0.905, RMSEA = 0.076, 90% CI (0.061, 0.091)], indicating that the hypothesized model was supported.

**Table 3:** Parcel-based SEM results of the proposed mediation model.

Parameter	B	SE	*β*	C. R.	p	95% CI	*R* ^2^
Structural paths							
Mindfulness → Emotional Intelligence	0.395	0.052	0.746	7.614	<0.001	–	0.557
Emotional Intelligence → Psychological Resilience	0.346	0.080	0.501	4.334	<0.001	–	0.338
Mindfulness → Psychological Resilience	0.038	0.037	0.103	1.023	0.306	–	–
Total effect							
Mindfulness → Psychological Resilience	0.175	–	0.477	–	<0.001	–	–
Direct effect							
Mindfulness → Psychological Resilience	0.038	–	0.103	–	0.306	–	–
Indirect effect							
Mindfulness → Emotional Intelligence → Psychological Resilience	0.137	–	0.374	–	<0.001	[0.065, 0.208]	–

B, Unstandardized coefficient; SE, Standard error; *β*, Standardized coefficient; C. R., critical ratio; CI, Confidence interval; *R*^2^, Squared multiple correlation.

Regarding the structural paths, mindfulness significantly and positively predicted emotional intelligence (*B* = 0.395, SE = 0.052, *β* = 0.746, C. R. = 7.614, *p* < 0.001). This finding indicates that higher levels of mindfulness were associated with higher levels of emotional intelligence. In addition, emotional intelligence significantly and positively predicted psychological resilience (*B* = 0.346, SE = 0.080, *β* = 0.501, C. R. = 4.334, *p* < 0.001), suggesting that individuals with greater emotional intelligence tended to report stronger psychological resilience. However, the direct effect of mindfulness on psychological resilience was not statistically significant (*B* = 0.038, SE = 0.037, *β* = 0.103, C. R. = 1.023, *p* = 0.306).

Examination of the mediation effects revealed that the indirect effect of mindfulness on psychological resilience through emotional intelligence was statistically significant [*B* = 0.137, *β* = 0.374, 95% CI (0.065, 0.208), *p* < 0.001]. Since the direct effect was non-significant while the indirect effect remained significant, the findings support a full mediation model. Accordingly, mindfulness appears to contribute to psychological resilience primarily through its positive association with emotional intelligence.

Finally, the model explained 55.7% of the variance in emotional intelligence (*R*^2^ = 0.557) and 33.8% of the variance in psychological resilience (*R*^2^ = 0.338), indicating moderate to substantial explanatory power.

Although the composite-score PROCESS analysis suggested partial mediation, the latent SEM analysis supported full mediation. This difference is theoretically plausible because SEM explicitly models measurement error, whereas regression-based PROCESS relies on observed total scores. Accordingly, SEM findings may provide a more conservative and construct-valid estimate of the indirect relationship.

Table 4 presents the comparison of mediation results obtained from the PROCESS analysis based on observed composite scores and the parcel-based structural equation modeling (SEM) analysis using latent variables. The findings indicated that both analytical approaches consistently supported a statistically significant indirect effect of mindfulness on psychological resilience through emotional intelligence, as the 95% confidence intervals in both models did not include zero.

**Table 4:** Comparison of mediation results across PROCESS and SEM analyses.

Method	Indirect effect (B)	95% CI	Direct effect	Mediation type
PROCESS	0.034	[0.020, 0.050]	0.097 (*p* < 0.001)	Partial mediation
SEM	0.137	[0.065, 0.208]	0.038 (*p* = 0.306)	Full mediation

However, the pattern of mediation differed across the two analytical frameworks. In the PROCESS analysis, the direct effect of mindfulness on psychological resilience remained statistically significant (*B* = 0.097, *p* < 0.001), indicating a partial mediation model. In contrast, the SEM analysis revealed that the direct effect became non-significant (*B* = 0.038, *p* = 0.306), supporting a full mediation model.

This discrepancy can be attributed to differences in the treatment of measurement error between the two approaches. While PROCESS relies on observed composite scores, SEM explicitly models latent variables and separates true-score variance from measurement error. As a result, SEM may provide a more precise estimation of structural relationships, leading to a more conservative interpretation of the direct effect.

Despite this difference, the consistency of the indirect effect across both analytical approaches provides strong evidence for the robustness of the proposed mediation mechanism. These findings suggest that emotional intelligence plays a central and stable mediating role in the relationship between mindfulness and psychological resilience, regardless of the analytical framework employed.

## Discussion

This study aims to determine how emotional intelligence mediates the relationship between psychological resilience and mindful awareness among physical education and sports teachers. The study, designed according to a correlational survey pattern, was conducted with the participation of 366 Turkish physical education and sports teachers.

The results of the present study indicated a significant positive association between mindful awareness and psychological resilience, thereby supporting Hypothesis 1. These findings are consistent with previous research conducted in both Turkish and international contexts (Karataş and Camadan, 2020; Aslan and Dalmış, 2025; Xu et al., 2016; Lee et al., 2021). In addition, meta-analytic evidence has demonstrated a significant positive relationship between mindfulness and psychological resilience across different populations (Liu et al., 2022).

From a theoretical perspective, mindfulness may facilitate more adaptive emotional and cognitive responses to stressful experiences by enhancing present-moment awareness and emotional regulation capacities. Such mechanisms may contribute to individuals’ ability to cope more effectively with adversity and maintain psychological resilience under challenging conditions. In the context of physical education and sports, the dynamic, interaction-intensive, and physically demanding nature of the profession may further underscore the importance of these psychological resources.

The present findings align with recent evidence suggesting that mindfulness plays a central role in regulating emotional responses to stress-inducing situations. In particular, the moderating effect of mindfulness in attenuating negative emotional reactions to perceived threats, as demonstrated among elite athletes (Solmaz and Yarayan, 2025), provides a meaningful framework for interpreting the direct and indirect effects observed in the current study. Supporting this perspective, research has highlighted the importance of cognitive-emotional resources in high-demand contexts. For example, Yarayan et al. (2025) demonstrated that mental energy is a significant determinant of flow state in football players, underscoring the role of psychological regulation processes in performance outcomes. From this perspective, mindfulness may contribute to psychological resilience by enhancing emotional awareness and regulation capacities (core components of emotional intelligence), which enable individuals to respond more adaptively to stress (Keng et al., 2011; Hülsheger et al., 2013).

A significant positive association was found between mindful awareness and emotional intelligence, thereby supporting Hypothesis 2. These findings are consistent with previous studies conducted in similar cultural contexts reporting positive relationships between mindfulness and emotional intelligence (Deniz et al., 2017; Cankurtaran and Şakiroğlu, 2020). For example, Deniz et al. (2017) reported that individuals with higher mindfulness levels also demonstrated stronger emotional awareness and regulation, whereas Cankurtaran and Şakiroğlu (2020) found that mindfulness-related attentional processes were positively associated with emotional intelligence dimensions.

From a theoretical perspective, mindfulness and emotional intelligence appear to share several overlapping psychological mechanisms, particularly those related to self-awareness, emotional monitoring, and emotional regulation (Schutte and Meynadier, 2024; Nataraj and Reddy, 2022). Mindfulness may contribute to individuals’ ability to recognize emotional experiences more accurately and respond to them in a more adaptive and less reactive manner, which may subsequently support the development of emotional intelligence–related capacities.

In educational settings, this relationship has also been supported by studies indicating that mindfulness and emotional intelligence are positively associated with teacher well-being, job satisfaction, and professional functioning (Aydogmus, 2023). Furthermore, mindfulness-based interventions have been shown to improve emotional regulation and emotional awareness capacities, which are considered central components of emotional intelligence (Demir and Gündoğan, 2018). Taken together, these findings suggest that mindfulness-related attentional and emotional processes may contribute to the development of emotional intelligence by strengthening individuals’ capacity to recognize, regulate, and manage emotional experiences effectively.

A significant positive association was found between emotional intelligence and psychological resilience, thereby supporting Hypothesis 3. This finding is consistent with previous research reporting that individuals with higher emotional intelligence tend to demonstrate stronger psychological resilience and more adaptive coping capacities under stressful conditions (İnaler and Turan, 2023; Kartol et al., 2024).

From a theoretical perspective, emotional intelligence may contribute to psychological resilience by facilitating more effective emotional awareness, regulation, and stress-management processes. Individuals with higher emotional intelligence are generally considered more capable of interpreting emotional experiences accurately and responding to challenging situations in a more adaptive manner. In educational settings, these capacities may play an important role in maintaining psychological functioning and professional well-being, particularly in occupations characterized by continuous interpersonal interaction and emotional demands (Soylu and Serin, 2017).

A growing body of research has consistently demonstrated a positive association between emotional intelligence and psychological resilience (Kartol et al., 2024). Individuals with higher emotional intelligence are generally considered better at recognizing, interpreting, and regulating emotional experiences, which may facilitate more adaptive coping responses under stressful conditions. In this respect, emotional intelligence may function as an important psychological resource supporting resilience, particularly in occupational environments characterized by continuous interpersonal interaction and emotional demands.

At the same time, previous studies suggest that the strength and nature of the relationship between emotional intelligence and psychological resilience may vary across different cultural and contextual settings (Miranda-Rochín et al., 2023), highlighting the complex interaction between individual characteristics and environmental conditions (Rao et al., 2024). In educational contexts, where teachers are frequently exposed to cognitive, emotional, and social stressors, emotional intelligence may help maintain psychological balance and adaptive functioning.

Furthermore, emotional intelligence has been positively associated with mindfulness, subjective well-being, emotional regulation, and life satisfaction (Griebel, 2015; Nataraj and Reddy, 2022; Martínez-Pérez et al., 2023). These findings support the theoretical assumption that mindfulness-related attentional and emotional processes may strengthen resilience capacities indirectly through mechanisms related to emotional intelligence. Specifically, Martínez-Pérez et al. (2023) reported that higher emotional intelligence was associated with greater psychological adaptation and well-being, whereas Nataraj and Reddy (2022) found positive relationships between emotional intelligence, mindfulness, and subjective well-being indicators.

Consistent with this theoretical framework, the present study demonstrated that mindfulness had a significant indirect association with psychological resilience through emotional intelligence, thereby supporting Hypothesis 4. Importantly, this mediating pathway was observed not only in the regression-based PROCESS analysis using observed composite scores but also in the complementary parcel-based structural equation modeling (SEM) analysis conducted within a latent-variable framework. While the PROCESS analysis suggested partial mediation, the SEM analysis indicated that the relationship between mindfulness and psychological resilience may operate primarily through emotional intelligence after accounting for measurement error.

These findings are generally consistent with previous studies reporting that emotional intelligence mediates the relationship between mindfulness and psychological adaptation outcomes (Martínez-Pérez et al., 2023). In particular, Martínez-Pérez et al. (2023) demonstrated that individuals with higher mindfulness and emotional intelligence reported better psychological adjustment and emotional well-being, suggesting that emotional intelligence may serve as an important mechanism linking mindfulness-related capacities to adaptive psychological functioning.

Similarly, research in athletic populations has shown that emotional intelligence may mediate the relationship between mindfulness-related processes and psychological resilience (Qi, 2025). More specifically, Qi (2025) reported that athletes with stronger mindfulness-related capacities demonstrated higher emotional intelligence levels, which subsequently contributed to greater resilience and more adaptive coping responses under performance-related stress. These findings support the assumption that mindfulness-related attentional and emotional processes may indirectly contribute to resilience via mechanisms related to emotional intelligence.

Taken together, the present findings suggest that emotional intelligence may function as a central psychological mechanism through which mindfulness-related capacities contribute to resilience-related adaptation. From a theoretical perspective, mindfulness may enhance present-moment awareness, emotional regulation, and cognitive flexibility, all of which have previously been associated with improved stress management and adaptive coping processes (Griebel, 2015; Nataraj and Reddy, 2022). In turn, individuals with higher emotional intelligence may be better able to accurately interpret emotional experiences, effectively regulate emotional reactions, and maintain psychological balance during stressful situations.

Accordingly, emotional intelligence appears to represent an important explanatory pathway linking mindfulness-related attentional processes with psychological resilience and broader adaptive functioning. Within educational and physically demanding occupational contexts such as physical education and sports teaching, these psychological capacities may play a particularly important role in sustaining emotional stability, coping effectiveness, and resilience under continuous interpersonal and occupational demands.

## Conclusion

This study provides evidence that mindful awareness is positively associated with psychological resilience among physical education and sports teachers, with emotional intelligence emerging as a robust mediating mechanism in this relationship. Findings from both composite-score and complementary latent-variable analyses suggest that individuals with higher levels of mindful awareness tend to demonstrate stronger emotional processing and regulation capacities, which in turn may facilitate more adaptive responses to stress and adversity.

By identifying emotional intelligence as an explanatory pathway, the study advances current understanding of how mindfulness-related capacities may translate into resilience within educational contexts characterized by high cognitive, emotional, and physical demands. These results are particularly relevant for physical education and sports teachers, for whom effective emotional regulation and psychological adaptability are essential for sustaining professional functioning, instructional quality, and occupational well-being.

From a practical standpoint, the findings support the integration of mindfulness-based approaches alongside emotional intelligence development within teacher education and in-service training programs. Interventions targeting both constructs may represent a more comprehensive strategy for strengthening psychological resilience and reducing the negative effects of occupational stress. In line with these implications, previous research has shown that mindfulness-based interventions and emotional intelligence training programs can improve psychological well-being, emotional regulation, and resilience in educational and occupational settings. Accordingly, structured programs focusing on these psychological resources may provide effective pathways for enhancing teachers’ adaptive capacities and coping skills.

The findings should be interpreted in light of the study’s cross-sectional design and reliance on self-report measures, which limit causal inference. Future research employing longitudinal, experimental, and intervention-based designs across diverse cultural and educational contexts is warranted to further clarify these mechanisms and determine potential causal pathways.

Overall, the present study highlights the interdependence of mindful awareness, emotional intelligence, and psychological resilience, positioning emotional intelligence as a central psychological process through which mindfulness-related capacities may support adaptive functioning in educators.

## Limitations

The present study has several limitations that should be considered when interpreting the findings. First, the research employed a cross-sectional correlational design. Therefore, although meaningful associations among the variables were identified, causal inferences cannot be drawn, and the findings should be interpreted with caution. Second, the sample consisted exclusively of Turkish physical education and sports teachers, which may limit the generalisability of the findings to other cultural contexts, age groups, and occupational populations.

Third, all variables were assessed using self-report measures, which may be subject to social desirability bias and common method variance, as participants’ responses were based on subjective perceptions. Although Harman’s single-factor test was conducted as an initial assessment, this technique has been criticized for its limited sensitivity and should not be considered a definitive test of common method bias (Podsakoff et al., 2003). Therefore, the potential influence of common method variance cannot be completely ruled out.

In addition, although the measurement instruments demonstrated acceptable levels of reliability overall, some psychometric indicators related to the emotional intelligence scale were below commonly recommended thresholds. Furthermore, several AVE values were below the conventional criterion of 0.50, which may indicate limited convergent validity at the latent construct level. Accordingly, findings involving emotional intelligence should be interpreted with some caution. Nevertheless, the consistency of the mediation pattern across both the composite-score PROCESS analysis and the complementary parcel-based SEM analysis provides partial support for the robustness of the overall findings.

Although the primary mediation analysis was initially conducted using composite scores within a regression-based framework, a supplementary parcel-based structural equation modeling analysis was also performed to evaluate the proposed relationships while accounting for measurement error. However, because parceling represents a more parsimonious latent modeling strategy, future studies with larger samples are encouraged to test the proposed model using full item-level SEM approaches.

Finally, although years of professional experience were included as a control variable to account for potential confounding effects, it did not emerge as a significant predictor in the model, suggesting that psychological resources such as mindfulness and emotional intelligence may operate relatively independently of tenure-related experiential factors.

## Recommendations

Given the limitations outlined, several recommendations can be made for future research. Firstly, studies conducted in different cultural contexts and across age and occupational groups would strengthen the generalisability of the findings. Furthermore, the effects of mindfulness-based programmes on emotional intelligence and psychological resilience should be tested empirically using experimental or quasi-experimental research designs. Longitudinal studies are needed to track changes in the relationships between variables over time. Furthermore, using mixed-methods designs to support quantitative findings with qualitative data will contribute to more comprehensive and in-depth interpretations. Finally, including additional variables such as social support, personality traits, stress levels, and academic achievement in the model will allow for a more holistic assessment of the relationships between mindfulness, emotional intelligence, and psychological resilience.

## Data Availability

The data analyzed in this study is subject to the following licenses/restrictions: The dataset used in this study is not publicly available due to ethical and privacy considerations. The data were collected from physical education and sports teachers under informed consent and include self-reported psychological measures. In order to protect participant confidentiality and comply with institutional ethical approval requirements, individual-level data cannot be shared in an open-access repository. However, anonymized and de-identified data may be made available by the corresponding author upon reasonable request for academic and non-commercial research purposes, provided that the requesting party agrees to maintain confidentiality and comply with applicable ethical standards. Requests to access these datasets should be directed to yusuf.buzdagli@erzurum.edu.tr.
