# Supplementary material for: The mediating role of emotional intelligence in the relationship between physical education and sports teachers’ mindfulness and psychological resilience
Source: Front Psychol. 2026 May 29;17:1815783. doi: 10.3389/fpsyg.2026.1815783 (PMC13259730; doi:10.3389/fpsyg.2026.1815783)
Supplement: Supplementary file 1 [file Table_1.docx]

**Manuscript Title:**
The Mediating Effect of Emotional Intelligence on the Relationship Between Physical Education and Sports Teachers' Mindfulness and Psychological Resilience

*This checklist has been completed in accordance with the STROBE (Strengthening the Reporting of Observational Studies in Epidemiology) guidelines.*

| **Section** | **Item No** | **Recommendation** | **Reported on Manuscript** |
| --- | --- | --- | --- |
| Title & Abstract | 1a | Indicate study design in title or abstract | Abstract |
| Title & Abstract | 1b | Provide informative and balanced abstract | Abstract |
| Introduction | 2 | Explain scientific background and rationale | Introduction |
| Introduction | 3 | State objectives and hypotheses | Introduction |
| Methods | 4 | Present key elements of study design | Study Model |
| Methods | 5 | Describe setting, locations, dates | Data Collection Procedure |
| Methods | 6a | Eligibility criteria for participants | Inclusion and Exclusion Criteria |
| Methods | 6b | Sources and methods of selection | Sampling and Recruitment Procedures |
| Methods | 7 | Clearly define variables | Study Model |
| Methods | 8 | Measurement methods and instruments | Data Collection Tools |
| Methods | 9 | Address potential sources of bias | Data Analysis |
| Methods | 10 | Explain study size determination | Research Group |
| Methods | 11 | Handling of quantitative variables | Data Analysis |
| Methods | 12a | Statistical methods | Data Analysis |
| Methods | 12b | Subgroup analyses | Not applicable |
| Methods | 12c | Missing data handling | Data Analysis |
| Methods | 12d | Sensitivity analyses | Not applicable |
| Methods | 13a | Participants at each stage | Data Analysis |
| Methods | 13b | Reasons for exclusions | Data Analysis |
| Results | 13c | Flow diagram | Not included |
| Results | 14a | Descriptive data | Results Table 1 |
| Results | 14b | Missing data information | Data Analysis |
| Results | 15 | Outcome data | Results Table 2 |
| Results | 16a | Main results | Results |
| Results | 16b | Category boundaries | Not applicable |
| Results | 16c | Risk estimates | Not applicable |
| Results | 17 | Other analyses | Results |
| Discussion | 18 | Key results summary | Discussion |
| Discussion | 19 | Study limitations | Limitations |
| Discussion | 20 | Interpretation of findings | Discussion |
| Discussion | 21 | Discuss generalisability of the findings | Limitations |
| Other | 22 | Give source of funding and role of funders | Funding |

**STROBE Checklist**
