# Supplementary material for: The mediating role of emotional intelligence in the relationship between physical education and sports teachers’ mindfulness and psychological resilience
Source: Front Psychol. 2026 May 29;17:1815783. doi: 10.3389/fpsyg.2026.1815783 (PMC13259730; doi:10.3389/fpsyg.2026.1815783)
Supplement: Supplementary file 2 [file Table_2.docx]

**Table S1. Parcel assignments used in the parcel-based SEM analysis**

| **Construct** | **Parcel Name in SEM** | **Items Included** |
| --- | --- | --- |
| Emotional Intelligence (TEIQue-SF) | Parcel 1 | Items 6, 13, 16, 18 |
| Emotional Intelligence (TEIQue-SF) | Parcel 2 | Items 2, 4, 10, 14 |
| Emotional Intelligence (TEIQue-SF) | Parcel 3 | Items 5, 11, 15, 19 |
| Emotional Intelligence (TEIQue-SF) | Parcel 4 | Items 1, 9, 12, 20 |
| Emotional Intelligence (TEIQue-SF) | Parcel 5 | Items 3, 7, 8, 17 |
| Mindfulness (MAAS) | Parcel 1 | Items 1, 2, 3, 4, 5 |
| Mindfulness (MAAS) | Parcel 2 | Items 6, 7, 8, 9, 10 |
| Mindfulness (MAAS) | Parcel 3 | Items 11, 12, 13, 14, 15 |
| Psychological Resilience (BRS) | Parcel 1 | Items 1, 2 |
| Psychological Resilience (BRS) | Parcel 2 | Items 3, 4 |
| Psychological Resilience (BRS) | Parcel 3 | Items 5, 6 |

**Table S2. Modification indices and theoretical justification for correlated residuals**

**Mindfulness (MAAS)**

| **Item Pair** | **Modification Index (MI)** | **Theoretical Justification** |
| --- | --- | --- |
| Item 7 - Item 10 | 83.418 | Strong overlap in attentional focus and present-moment awareness |
| Item 12 - Item 15 | 26.931 | Conceptual similarity in awareness-related processing |

**Emotional Intelligence (TEIQue-SF)**

| **Item Pair** | **Modification Index (MI)** | **Theoretical Justification** |
| --- | --- | --- |
| Item 13 - Item 15 | 35.263 | Items reflect similar emotional regulation processes |
| Item 1 - Item 3 | 26.476 | Items share similar emotional perception content |
| Item 17 - Item 20 | 28.989 | Items represent overlapping aspects of emotional self-regulation |

**Psychological Resilience (BRS)**

| **Item Pair** | **Modification Index (MI)** | **Theoretical Justification** |
| --- | --- | --- |
| Item 1 - Item 4 | 49.598 | Overlap in resilience-related coping and recovery processes |
| Item 1 - Item 3 | 34.919 | Conceptual similarity in resilience perception |
| Item 2 - Item 3 | 15.014 | Shared variance in recovery-related responses |

**Note:** Parcel construction procedures differed across scales according to their structural characteristics. Sequential parceling was used for the MAAS and BRS to preserve the unidimensional structure of the scales, whereas a domain-representative parceling approach was applied for the TEIQue-SF based on conceptual similarity and factor loadings.
